# Quantification of the Intracellular Life Time of Water Molecules to Measure Transport Rates of Human Aquaglyceroporins

**DOI:** 10.1007/s00232-017-9988-4

**Published:** 2017-09-15

**Authors:** Madelene Palmgren, Malin Hernebring, Stefanie Eriksson, Karin Elbing, Cecilia Geijer, Samo Lasič, Peter Dahl, Jesper S. Hansen, Daniel Topgaard, Karin Lindkvist-Petersson

**Affiliations:** 1 Department of Chemistry and Molecular Biology, University of Gothenburg, Box 462, 405 30 Göteborg, Sweden; 2 Department of Experimental Medical Science, Lund University, BMC C13, 221 84 Lund, Sweden; 3 Physical Chemistry, Lund University, P.O.B. 124, 22100 Lund, Sweden; 4 CR Development, AB, Naturvetarvägen 14, 22362 Lund, Sweden

**Keywords:** Aquaporin, Aquaglyceroporin, Water transport, NMR, *P. pastoris*

## Abstract

Orthodox aquaporins are transmembrane channel proteins that facilitate rapid diffusion of water, while aquaglyceroporins facilitate the diffusion of small uncharged molecules such as glycerol and arsenic trioxide. Aquaglyceroporins play important roles in human physiology, in particular for glycerol metabolism and arsenic detoxification. We have developed a unique system applying the strain of the yeast *Pichia pastoris*, where the endogenous aquaporins/aquaglyceroporins have been removed and human aquaglyceroporins AQP3, AQP7, and AQP9 are recombinantly expressed enabling comparative permeability measurements between the expressed proteins. Using a newly established Nuclear Magnetic Resonance approach based on measurement of the intracellular life time of water, we propose that human aquaglyceroporins are poor facilitators of water and that the water transport efficiency is similar to that of passive diffusion across native cell membranes. This is distinctly different from glycerol and arsenic trioxide, where high glycerol transport efficiency was recorded.

## Introduction

Aquaporins (AQPs) are transmembrane proteins that are represented throughout all kingdoms of life, including animals and plants as well as in lower organisms such as yeast and bacteria. Their primary function is to facilitate water and glycerol transport across cell membranes (Verkman 2012). The aquaporin family is commonly divided into three sub-groups, the orthodox aquaporins (sole water facilitators), the aquaglyceroporins (that facilitate the transport of solutes such as glycerol, arsenic trioxide and urea), and the superaquaporins. There are 13 human aquaporins abbreviated AQP0-12, which are widely distributed in specific cell types in many organs and tissues (Day et al. 2014). AQP3, 7, 9, and 10 belong to the aquaglyceroporin sub-group, and in recent years, several reports suggest that the human aquaglyceroporins are essential players in human health and disease (Hara-Chikuma and Verkman 2008b; Maeda 2012). Commonly, the aquaglyceroporins are stated to have dual permeability, both for water and solutes such as glycerol (Laforenza et al. 2016). The transport specificity of the aquaglyceroporins is well-documented in the literature, frequently using either *Xenopus laevis* oocytes to measure the water transport rates, or artificial membranes creating liposomes with inserted proteins. In both these systems, the water transport is indirectly measured by detecting the swelling/shrinkage of the oocytes/liposomes upon an applied change in osmolality, and water transport is quantified in terms of the osmotic water permeability coefficient. Here, we apply a more direct method for studying water exchange over biological membrane by using nuclear magnetic resonance (NMR), which allows for the transport mechanisms to be studied in equilibrium conditions without applying an osmotic gradient (Eriksson et al. 2017).

We established a unique approach to measure transport of water, glycerol, and arsenic trioxide [As(III)] in a native cell membrane using the yeast *Pichia pastoris*. In this approach, human AQP3, AQP7, and AQP9 are highly expressed in an AQP deletion strain of *P. pastoris*, where both endogenous aquaporins (Aqy1 and Agp1) have been removed by standard cloning strategies. Spheroplasts of these strains have AQP-containing lipid bilayers without requiring elaborate protein purification schemes or need of exchanging detergent micelle-stabilized membrane proteins into the lipid bilayers, necessary in liposome-based approaches. We applied the diffusion NMR method that enables quantification of the rate of water exchange between intracellular and extracellular compartments on a millisecond time-scale (Eriksson et al. 2017). Interestingly, in our system, when human aquaglyceroporins are in their native environment (lipid bilayer) and the water transport rate is measured by the non-invasive NMR method, the water transport capability of the human aquaglyceroporins are not significantly different from the passive water diffusion across the plasma membrane of cells without aquaglyceroporins expressed. Still, all aquaglyceroporins investigated significantly facilitate the transport of glycerol and As(III) in the same system. Thus, we suggest that human aquaglyceroporins are poor water facilitators compared to orthodox aquaporins, and that their main function in the body is to facilitate transport of other solutes such as glycerol and arsenic trioxide.

## Materials and Methods

### Yeast Strains

*Pichia pastoris* strains used in this study derives from the X33 strain (commercially available from Thermo Fisher, Life Technologies, USA). The protein encoded by gene PAS_chr4_0784 in *P. pastoris* GS115 was identified with 35% sequence identity using Fps1 as query in a BLASTp. This protein was named Aquaglyceroporin 1 (Agp1). A double-deletion strain *P. pastoris* GS115 *aqy1Δ::HIS4 agp1Δ::NatMX* was generated by inserting a deletion cassette by homologue recombination into *P. pastoris* GS115 *aqy1Δ::HIS4* (Fischer et al. 2009). The double-cloning approach used for deleting *AGP1* was previously described by Ternes and co-workers (Ternes et al. 2011). A ~670 bp fragment upstream of *AGP1*-containing restriction sites for XbaI and NotI and a ~800 bp downstream fragment reaching into the *AGP1* gene containing NotI and XbaI restriction sites were amplified from genomic DNA by PCR (primer 1 + 2 and 3 + 4, respectively, Table 1). Both fragments were simultaneously ligated into the NotI site of a pPICZ B vector. The antibiotic resistance gene nourseothricin was cut out from the pSLnat plasmid (Ternes et al. 2011) and inserted into the NotI site of pPICZB plasmid flanked by upstream and downstream regions to the *AGP1* gene. The construct was confirmed by sequencing. The deletion cassette was amplified by PCR (primer 5 and 6, Table 1) and transformed into *P. pastoris* GS115 *aqy1Δ::HIS4* (Cregg 2007). Transformed cells were plated on YPD-agar plates including 1% *w*/*v* yeast extract, 2% *w*/*v* peptone and 2% *w*/*v* agar, 2% *w*/*v* glucose, and grown for 2 days at room temperature before they were replica plated onto YPDS-agar plates (YPD-agar plates + 1 M sorbitol) + Nourseothricin (clonNAT) (15 µg/ml) (GoldBio, USA). Plates were incubated at 30 °C for 3-4 days. Transformants were re-streaked for single colonies on YPD-agar plates + clonNAT (15 µg/ml). Successful deletion of the *AGP1* gene was verified by genomic PCR using 5 different primer pairs (primer 1 + 10, 1 + 11, 1 + 4, 7 + 8, and 9 + 4, Table 1).

**Table 1:** Primers used for cloning. Nucleic acid sequences for primers used for creating the *AGP1* deletion in *P. pastoris* GS115 *aqy1Δ* strain

Primer#	Primer sequence (5′-3′ - direction)
1	CAGTGAATTCGGAAGGTCAATCTACTACACGTG
2	GACTGCGGCCGCGCAACTTTCACCAGCTTAGC
3	CATAGCGGCCGCTTGTGGCTTCTGCGTTCC
4	GGTGGCAGTTCTAGATCGAGG
5	AAGGTTTCAGGACCTGTTGCT
6	TATTCTCGTCTCCTATTGGCG
7	CACAAATCCAAGAGACTGAAGAC
8	CGATAGCTTGAATGTAAGTACCG
9	AGCGCTTGTTCTTGAAGAGT
10	ACTCTTCAAGAACAAGCGCT
11	CGAGTCTTCAGTCTCTTGGAT

### Protein Expression and Western Blot Analysis

The human aquaporins AQP1, AQP3, AQP7, and AQP9 with a His_6_ purification tag were cloned into pPICZB vector using restriction enzyme EcoRI and XbaI. To identify clones that express the protein of interest, AQP transformants were spotted on YPD-agar plates supplemented with increasing zeocin concentrations (0, 0.5, 1.0, and 2.0 mg/ml) (Thermo Fisher, Life Technologies, USA). The five most zeocin-resistant clones of each AQP construct were further analyzed for expression levels. Cells were pre-grown in buffered glycerol complex medium (BMGY: 1% *w*/*v* yeast extract, 2% *w*/*v* peptone, 100 mM potassium phosphate pH 6.0, 1.34% *w*/*v* yeast nitrogen base, 0.4 mg/L biotin, 1% *v*/*v* glycerol) for 24 h, and protein production was induced with methanol to a final concentration of 0.25% *v*/*v* for 6 h (for AQP3, AQP7, AQP9) or 1 h (for AQP1). Cells were harvested at 2000×*g* for 5 min and washed in 20 mM HEPES pH 7.5. Plasma membranes were purified using the protocol of Panaretou and Piper (2006). Approximately, 300-μl cell pellet was harvested and re-suspended in an equal volume of breaking buffer (20 mM Tris–HCl pH 7.5, 0.4 M sucrose, 4 mM EDTA, 2 mM DTT) containing protease inhibitor (Roche Diagnostics) and glass beads. Cells were broken by Fast Prep (MP Biomedical, USA), cell debris was removed at 600×*g* for 10 min at 4 °C, and the total membrane was collected by centrifugation at 21,000×*g* for 30 min at 4 °C. Total membranes were re-suspended in membrane resuspension buffer (10 mM Tris–HCl pH 7.5, 2 mM EDTA). A sucrose gradient containing equal volumes of 2.25, 1.65, and 1.1 M sucrose in 10 mM Tris–HCl pH 7.5 and 2 mM EDTA was overlaid with the total membrane sample and spun overnight in a SW Ti60 centrifuge at 40,000 rpm at 4 °C. The plasma membrane fraction was removed from the 2.25/1.65 M interphase and diluted five times into membrane resuspension buffer and spun for 30 min at 20,000×*g* at 4 °C. Pellet was re-suspended in plasma membrane resuspension buffer (20 mM Tris–HCl pH 7.5, 150 mM NaCl, 10% *v/v* glycerol) with protease inhibitor. Purified plasma membrane samples were resolved on NuPAGE 4–12% Bis–Tris (Thermo Fisher, Life Technologies, USA) gels and blotted onto nitrocellulose membranes (Hoefer Inc, USA). Membranes were blocked with fish gelatin blocking buffer (10 mM Tris–HCl pH 7.5, 150 mM NaCl, 2% *w/v* Fish Gelatin, 1% *w/v* Ovalbumin). Antibodies were incubated with blocked membrane in 1:1 blocking buffer and 20 mM Tris–HCl pH 7.5, 100 mM NaCl with 0.1% *v/v* Tween-20 (TBS-T). The plasma membrane marker, Pma1, was detected using rabbit-anti-Pma1 antibody (Santa Cruz Biotechnology, USA; 1:1,000, #sc-33735) at 4 °C overnight and the His-tag was detected with the mouse-anti-His antibody (Sigma-Aldrich, USA; 1:5000) for 45 min at room temperature. Donkey-anti-mouse 680 nm (1:10,000) and donkey-anti-rabbit 800 nm (1:10,000) were used as secondary antibodies, and incubated for 45 min at room temperature (LI-COR, USA). Fluorescent signals were detected using Odyssey FC (LI-COR, USA). The endoplasmic reticulum (ER) marker, Sec61, antibody was used at 1:1000 and incubated over night at 4 °C. The signal was detected using an anti-rabbit horseradish peroxidase antibody and enhanced chemiluminescence (ECL). The signal was detected by the Gel DocTMsystem (BIO-RAD, USA).

### Diffusion NMR

Cells were grown and protein production induced as described above. Cells were harvested and washed with 20 mM HEPES pH 7.5, re-suspended in a 1:1 *v/v* solution with buffer, and transferred to 5-mm disposable NMR tubes and centrifuged at 1000×*g* for 10 min to achieve a pellet with high concentration of cells. The tubes were kept on ice until measurement. The NMR diffusion measurements were done at 0 °C for AQP1 and *aqy1Δagp1Δ* induced for 1 h and at 20 °C for strain AQP3, 7, and 9 and *aqy1Δagp1Δ* induced for 6 h. According to Eriksson et al., the temperatures and induction times were chosen so that the same set of mixing times (*t*_m_) allowed a precise quantification of exchange for all strains, *aqy1*Δ*agp1*Δ, *aqy1*Δ*agp1*Δ + hAQP1, and *aqy1*Δ*agp1*Δ + AQP3,7,9 (Eriksson et al. 2017). Measurements were performed on a Bruker 200 MHz Avance-II spectrometer with a DIF-25 gradient probe capable of *z*-gradients up to 9.6 T/m. The NMR method used is described in Eriksson et al. (Eriksson et al. 2017) and identical experimental parameters were used here. The method combines a filter exchange spectroscopy (FEXSY) pulse sequence with a pulsed-gradient spin-echo (PGSE). The FEXSY experiment consists of two diffusion-encoding blocks separated by a mixing time, *t*_m_, and is specifically sensitive to exchange. The PGSE pulse sequence was applied with varying echo time, *t*_E_, to correlate the intra- and extracellular diffusion coefficients and *T*_2_-relaxation times and was used to compensate for differences in *T*_2_-relaxation between the intra- and extracellular water. The intra- to extracellular exchange rate was obtained by a constrained global fitting of both datasets.

### Stopped Flow Measurements

Cells were isolated at 2000×*g* for 5 min and washed three times in 20 mM HEPES pH 7.5 and 1.2 M Sorbitol, and re-suspended to a final absorbance at A_600_ of 3. In the Stopped Flow Machine (SFM) procedure, cells were subjected to a hyperosmotic shock (20 mM HEPES pH 7.5 and 1.8 M Sorbitol) with a mixing rate of 7 ml/s in a ratio of 1:1 to a final volume of 148 µl. The cell response (shrinkage) was monitored as increased light-scattering intensity at 435 nm at an angle of 90° using a SFM-20 and MOS-450 spectrometer (BioLogic).

### Glycerol Transport Measurement

Washed cells were re-suspended to an approximate density of 30 mg/ml, and subsequently the absorbance at A_600_ was measured and precisely adjusted for all strains. Carbonyl Cyanide m-Chlorophenylhydrazone (CCCP) was added to a final concentration of 100 µM to the cell suspension to inhibit uptake of glycerol by active transporters, and equilibrated with the cells at 30 °C for 10 min prior to each measurement. Uptake studies were initiated by the addition of a glycerol mix (300 mM glycerol and 40 µM [^14^C] glycerol (142.7 mCi/mmol; Perkin Elmer)). The reaction was stopped by transferring the whole mixture to a pre-filled funnel with 5 ml pre-chilled 20 mM HEPES, pH 7.5, 500 mM glycerol. Cells were then washed and disintegration per minutes (dpm) was recorded as previously described (Tamas et al. 1999). The measured signal in dpm was converted to nmol glycerol per OD unit and plotted over time. For measurement carried out at pH 6.0, 20 mM MES pH 6.0 was used instead of 20 mM HEPES pH 7.5.

### As(III) Transport and Phenotype Assay

For each individual measurement, 10 OD units of washed cells were re-suspended in 20 mM HEPES pH 7.5 and incubated at 30 °C for 5–10 min prior measurement. Uptake studies were initiated by the addition of NaAsO_2_ in 1:1 (*v/v*) ratio with a final concentration of 100 mM. The reaction was stopped by transferring the cell suspension to cold 20 mM HEPES buffer pH 7.5 and collected on a Whatman^®^ glass microfiber filter Grade GF/C and washed twice in cold 20 mM HEPES buffer. Cells were re-suspended in water and disrupted by boiling. Cell debris was sedimented at 10,000×*g* for 10 min. Sodium arsenite-containing supernatants were sent to analysis by inductively coupled plasma mass spectrometry (ICP-MS). For the growth assay, cells were cultivated and protein expression was induced (as described above). Cells were diluted in 20 mM HEPES pH 7.5 to an absorbance at A_600_ of 0.2. Five microliters from 10 times dilution series were spotted on agar plates containing buffered methanol complex medium (BMMY: 1% *w*/*v* yeast extract, 2% *w*/*v* peptone, 100 mM potassium phosphate pH 6.0, 1.34% *w*/*v* yeast nitrogen base, 0.4 mg/l biotin, 0.5% *v*/*v* methanol, 2% *w*/*v* agar) with and without NaAsO_2_ (0 and 600 µM) and incubated at 30 °C for 3 days.

### Statistical Analysis

Comparisons between multiple groups were done in GraphPad Prism by one-way ANOVA followed by Tukey’s multiple comparisons test and the null hypothesis was rejected at the 0.05 confidence level.

## Results and Discussion

The yeast *P. Pastoris* has been successfully used for heterologous protein production and, particularly, in recent years also for overexpressing transmembrane proteins. Thus, *P. pastoris* is a suitable system for measuring the activity of human transmembrane proteins. In this study, we aim at pinpointing the detailed specificity of the human aquaglyceroporins. The genome for *P. pastoris* GS115 has been public since 2009 (De Schutter et al. 2009) and contains two aquaporin genes encoding the previously characterized Aqy1 (Fischer et al. 2009) and a second putative aquaglyceroporin, Agp1 (AquaGlyceroPorin1).

### A Novel System to Study Substrate Specificity of Human Aquaglyceroporins

**Fig. 1:**
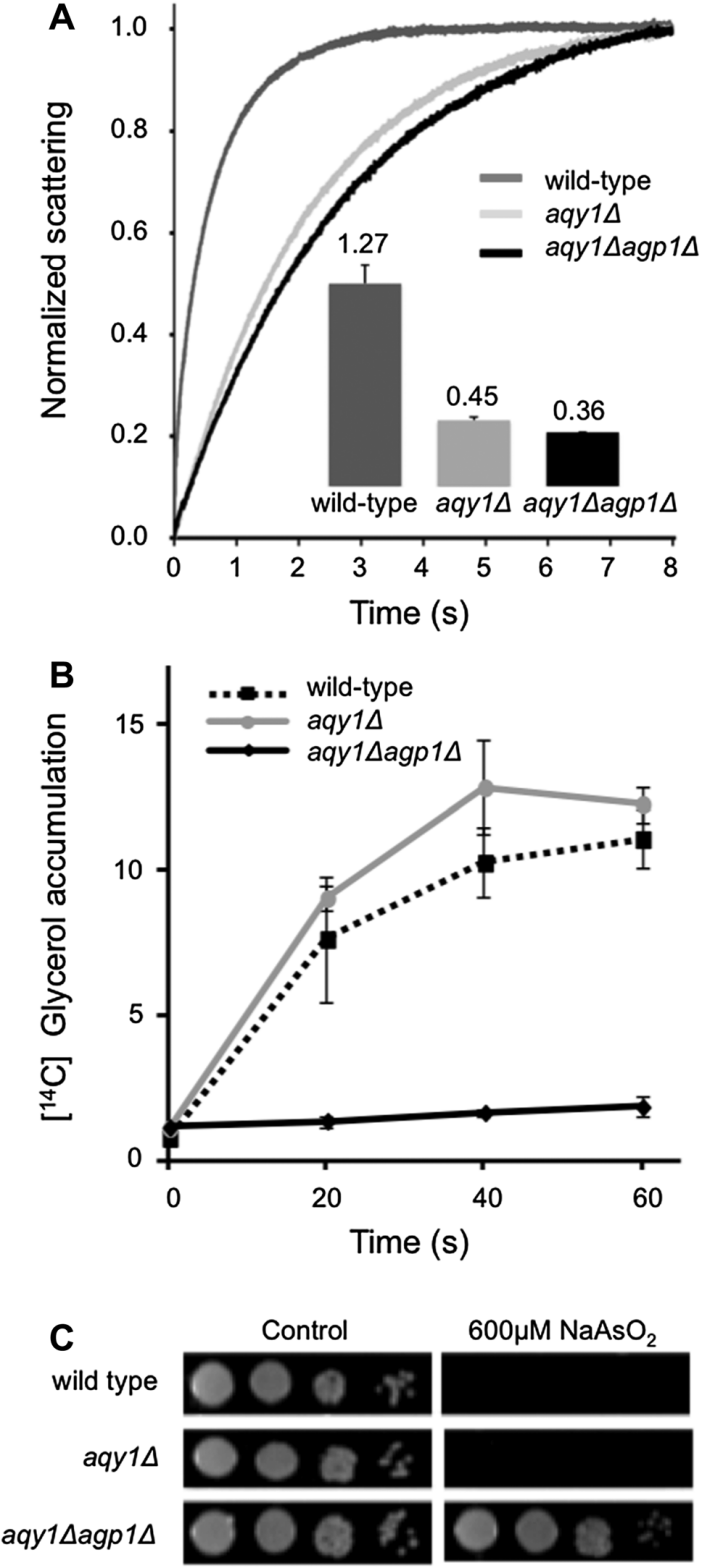
A novel yeast strain of *P. pastoris* that is impermeable for water, glycerol, and arsenic trioxide. Comparison of solute permeation in different strains of *P. pastoris*. **a** Cells were subjected to a hyperosmotic shock causing outward movement of water. Normalized Light Scatter Intensity (NLSI) was plotted over time (s). The rate constant, *k*, was obtained by curve fitting using the equation: $y = A_{1} \cdot e^{{ - k_{1} \cdot t}} + A_{2}$ (*A*_1_ Amplitude, *k* rate constant, *t* time, *A*_2_ = offset), and are shown as bars (inset), values are mean of duplicates, and error bars denote SD. **b** Glycerol accumulation per OD unit measured as a function of time after addition of [^14^C] glycerol. Values shown are mean normalized to the mean 0-value in each experiment and error bars denote standard deviation (SD). **c** Cells were spotted on Control media (BMMY, Buffered Methanol complex Media) with and without NaAsO_2_ in 10 times dilution series to monitor As(III) sensitivity. A representative image is shown

To enable functional studies of human aquaglyceroporins, a *P. pastoris* strain deficient in endogenous aquaporins, Aqy1 and Agp1 proteins, was generated. *P. pastoris* has previously been used to measure water transport rate of heterologously expressed aquaporins, however, both the endogenous aquaporins of *P. pastoris* were not removed in those studies, which may impact the results (Azad et al. 2009; Fischer et al. 2009). To confirm that the transport abilities typical for both aquaporins and aquaglyceroporins are disrupted in our strain, water, glycerol, and As(III) transport efficiency was measured in a *P. pastoris* wild-type strain (X33), single-deletion strain *P. pastoris GS115 aqy1Δ* (where the orthodox aquaporin is deleted) (Fischer et al. 2009) and a new double-deletion strain *P. pastoris GS115 aqy1Δagp1Δ* (where both *AQY1* and aquaglyceroporin, *AGP1* are deleted) (Fig. 1). The water transport rates were assayed using the standard stopped-flow light-scattering technique, where spheroplasts of the three different strains (wild type, *aqy1Δ*, *aqy1Δagp1Δ*) were subjected to a hyperosmolality solution and the concomitant change in light scattering was measured as a function of time (Fig. 1a). The wild-type strain, containing both aquaporins, had a rate constant around three times higher the values of both the *aqy1Δ* strain and the *aqy1Δagp1Δ* strain, suggesting that it has roughly three times higher water transport (Fig. 1a). Marginal differences in water transport rates are observed between the two deletion strains, which are interpreted as Agp1 is not considerably contributing to the water efflux and hence has poor water transporting capability (Fig. 1a). Glycerol uptake efficiency was measured as accumulation of radiolabeled glycerol over time (Fig. 1b). The wild type and the *aqy1Δ* strain readily accumulated glycerol, while the *aqy1Δagp1Δ* strain did not, suggesting that Agp1 is a glycerol conducting channel. To test whether arsenic trioxide, a highly toxic compound, is a potential substrate for Aqy1 and/or Agp1, cells from the three different strains were spotted on As(III)-containing plates (Fig. 1c). In this assay, cells expressing efficient As(III) facilitators will not survive. Cell growth was clearly affected in both wild type and *aqy1Δ* strains, while the *aqy1Δagp1Δ* strain showed tolerance towards arsenic trioxide. This suggests that both the wild-type strain and the *aqy1Δ* strain take up As(III), while the *aqy1Δagp1Δ* strain cannot, and hence Agp1 is likely responsible for the uptake. Taken together, by deleting *AQY1* and *AGP1* in *P. pastoris*, we have generated a novel system where heterologous aquaporins can be expressed and their substrate specificity for water, glycerol, and As(III) can be evaluated.

### The Human Aquaglyceroporins are Poor Water Facilitators

**Fig. 2:**
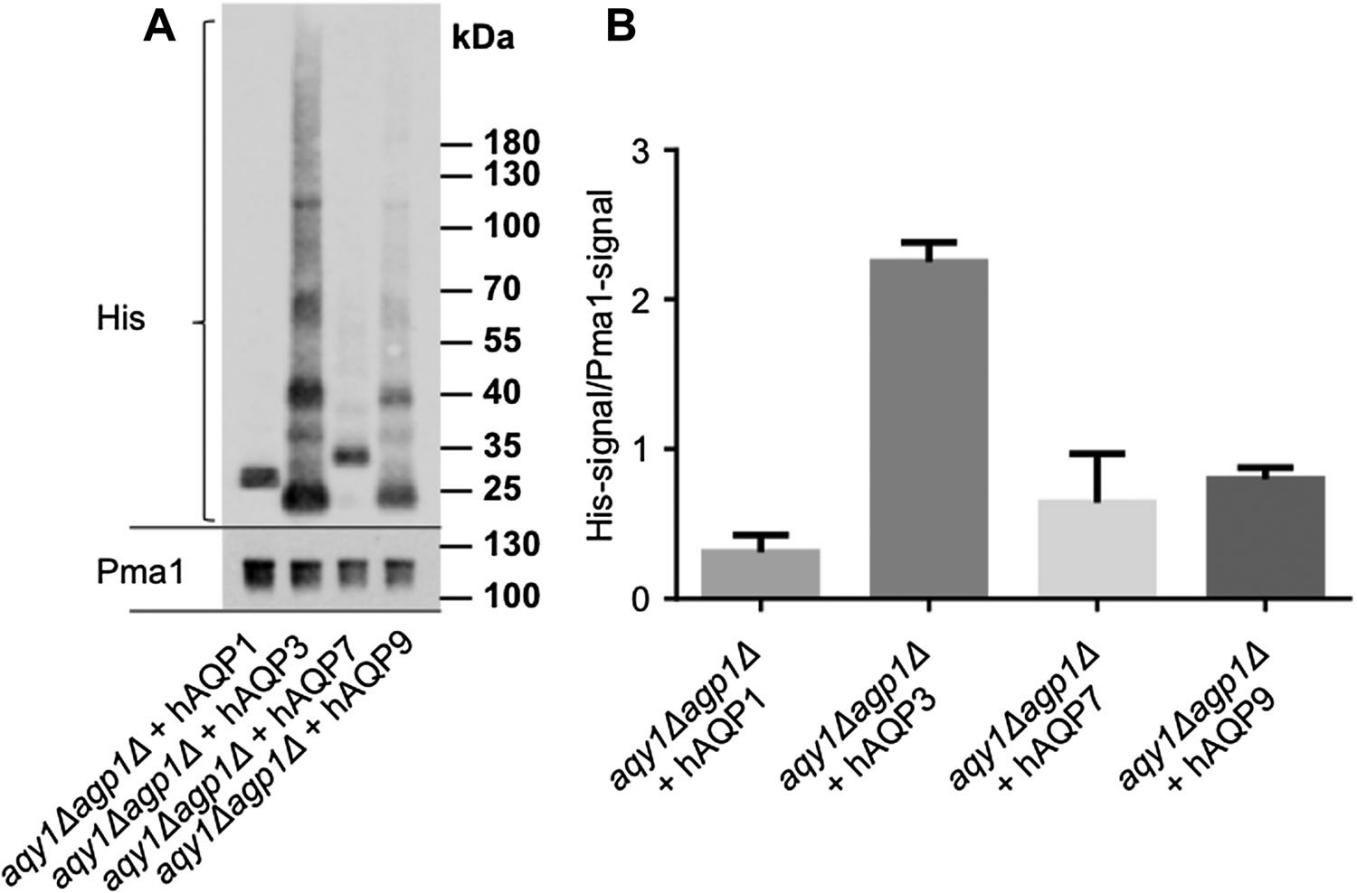
Quantification of aquaglyceroporins levels in the plasma membrane. **a** Quantitative Western blot analyses of aquaglyceroporins present in the plasma membrane. Protein content in plasma membrane fractions were analyzed by SDS-PAGE and Western blotting detecting Pma1 membrane marker and the polyhistidine-tag of the AQPs. Note that the typical aquaporin migrates in the gel as different mono/oligomers. **b** Quantification of the aquaglyceroporins present in the plasma membrane (His-signal divided by Pma1-signal), values are mean, and error bars denote SD

**Fig. 3:**
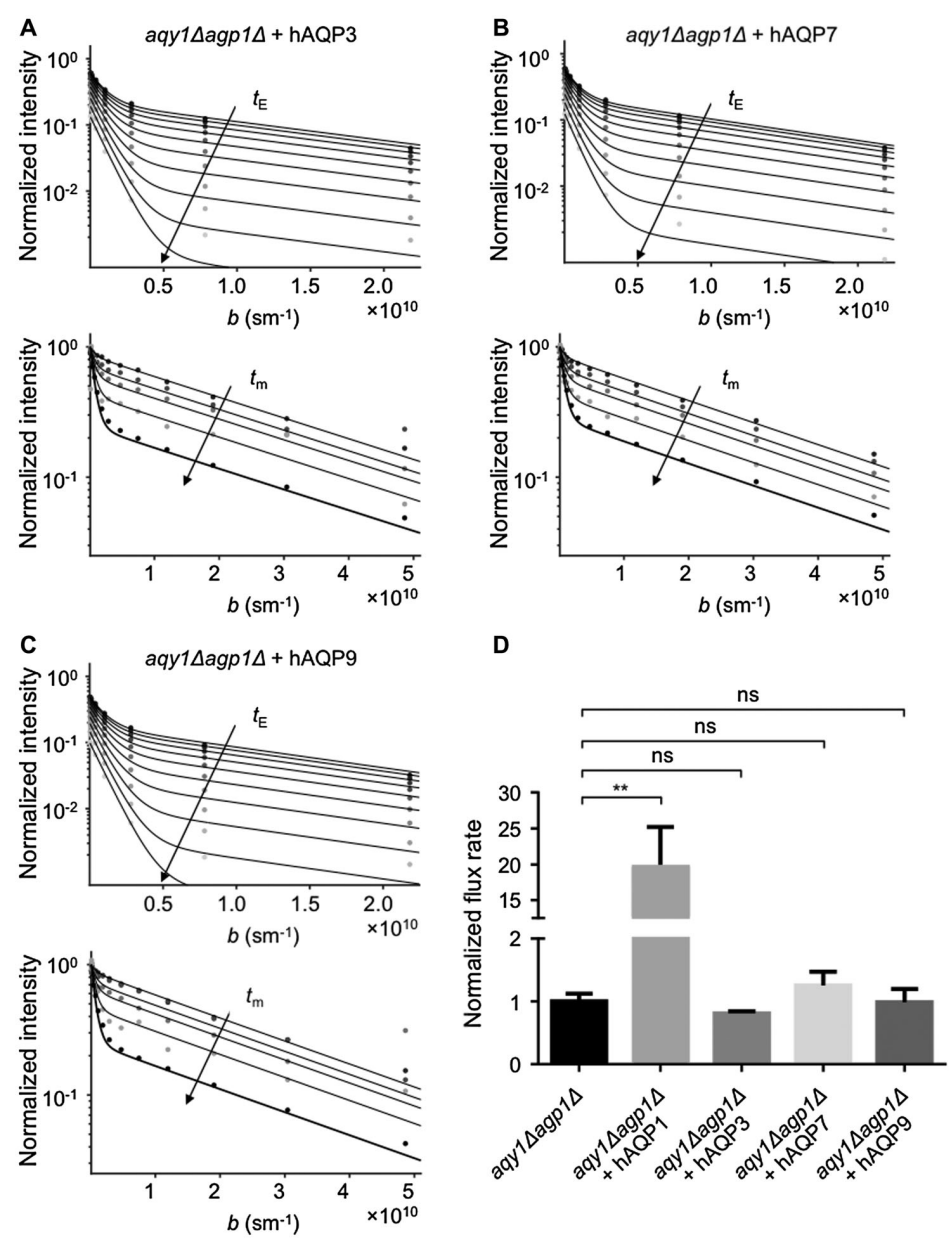
Diffusion NMR method used to measure water exchange in cells. Water transport efficiency was measured for the different strains using the NMR diffusion technique. **a**–**c** Examples of results from NMR diffusion experiments showing normalized signal intensity for (**a**) *aqy1Δagp1Δ* + hAQP3, (**b**) *aqy1Δagp1Δ* + hAQP7, and (**c**) *aqy1Δagp1Δ* + hAQP9 as a function of diffusion weighting *b*. The upper graph shows signal attenuation curves from the PGSE experiment for increasing echo times, *t*_E_, indicated by arrow. The lower graph shows signal attenuation curves from the FEXSY experiment where arrow indicates increasing mixing times, *t*_m_. (**d**) Statistical significance was established by one-way ANOVA (*F*(4,10) = 11.27, *p* = 0,001), and Tukey’s multiple comparisons test rendered statistical significant differences (*p* < 0.01) exclusively between *aqy1Δagp1Δ* + hAQP1 and the other strains (*aqy1Δagp1Δ*, *aqy1Δagp1Δ* + hAQP3, *aqy1Δagp1Δ* + hAQP7, *aqy1Δagp1Δ* + hAQP9), but non-significant differences in all other comparisons. The *aqy1Δagp1Δ* + hAQP1 strain was analyzed at 0 °C together with *aqy1Δagp1Δ* control, while the other strains were analyzed at 20 °C. Values are mean values normalized to *aqy1Δagp1Δ* in each experiment (ki/ki_*aqy1Δagp1Δ*_) and error bars denote SEM

**Fig. 4:**
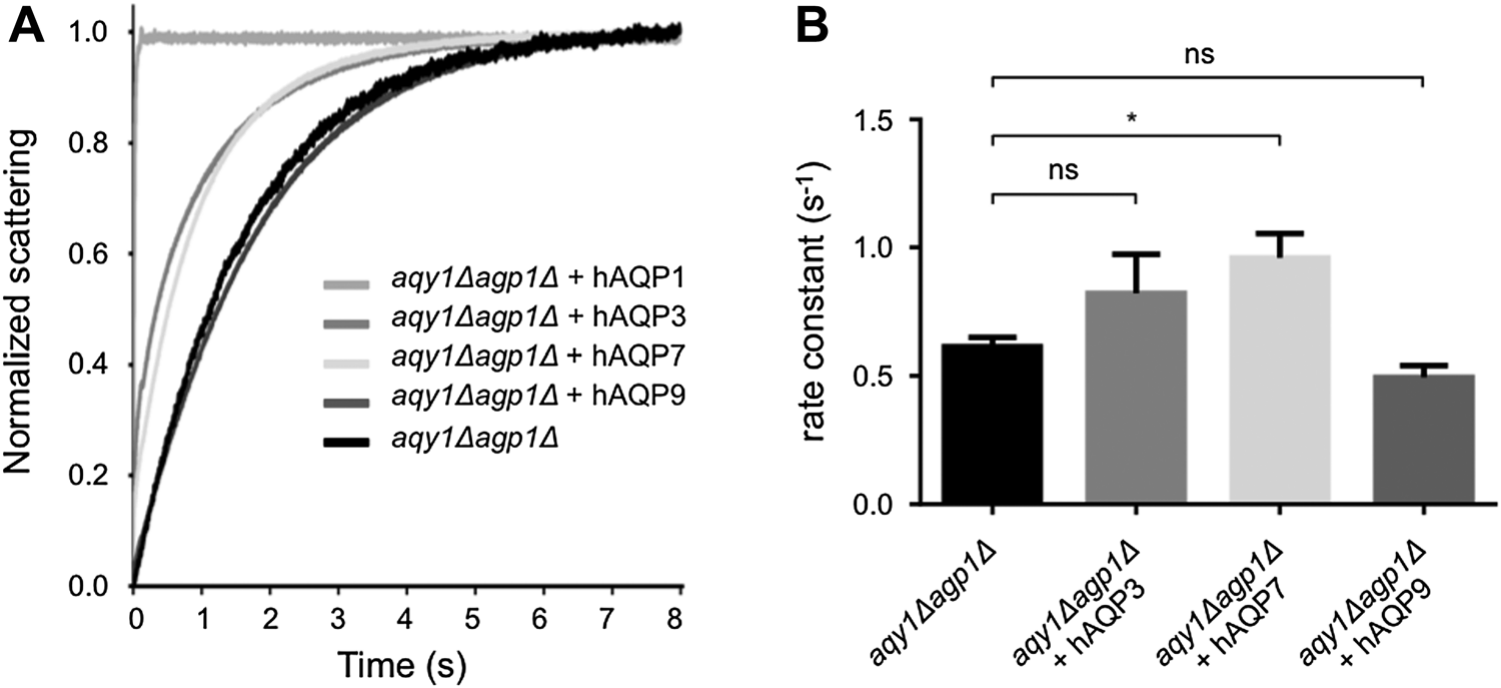
Applying SFM to measure cell shrinkage as indicative for water efflux. **a** Cells were subjected to a hyperosmotic shock causing outward movement of water. Normalized Light Scatter Intensity (NLSI) was plotted over time (s). **b** Water exchange rate constant (*k*) for *aqy1Δagp1Δ*, *aqy1Δagp1Δ* + hAQP3, *aqy1Δagp1Δ* + hAQP7, *aqy1Δagp1Δ* + hAQP9, values are mean of triplicates, and error bars denote SEM. Statistical significance was investigated with one-way ANOVA (*F*(3,8) = 14.38, *p* < 0.01), and Tukey’s multiple comparisons test rendered statistical significant difference (*p* < 0.05) between *aqy1Δagp1Δ* and *aqy1Δagp1Δ* + hAQP7 and non-significant differences comparing *aqy1Δagp1Δ* with *aqy1Δagp1Δ* + hAQP3 and *aqy1Δagp1Δ* + hAQP9

To investigate the transport specificity of the human aquaglyceroporins for water, glycerol, and As(III), hAQP1, hAQP3, hAQP7, and hAQP9 were expressed in the double-deletion strain of *P. pastoris* (*aqy1Δagp1Δ)* (Fig. 2a). The levels of protein expression in the plasma membrane were quantified using Western blotting of plasma membrane fractions, detecting the histidine purification tag fused to the respective aquaporin (His) and a plasma membrane marker (Pma1), to relate aquaporin levels to the plasma membrane purity of the fraction samples (Fig. 2a, b). Previously, the *X. laevis* oocyte expression system has been widely used to investigate the permeability of water for human AQP3, AQP7, and AQP9, and all of them facilitated the transport of water in that system (Boury-Jamot et al. 2006; Ishibashi et al. 1998; Kondo et al. 2002). Along those lines, human AQP3 has also been shown to facilitate the transport of water in liposomal model membranes (Muller-Lucks et al. 2013). In these previous studies, water transport was measured indirectly as the water transport activity is estimated by the volume change of the oocyte/liposome after a rapid change in osmolality of the surrounding medium. We have applied an NMR diffusion technique to measure the actual rate of water exchange between intracellular and extracellular space across a native cell membrane. The intracellular lifetime of water molecules was determined by quantifying the mean squared displacement of water molecules as a function of diffusion time on a millisecond time-scale (Eriksson et al. 2017). In contrast to previous reports, our NMR results, where the intra- to extracellular exchange rate was obtained by a constrained global fitting, show that aquaglyceroporins are poor water facilitators, as compared to the orthodox aquaporin AQP1 (Fig. 3). Their transport activity was measured to be in the same range as passive water diffusion over the membrane (Fig. 3d). To compare the results from the novel NMR method with standard indirect analysis of water permeability using the stopped-flow light-scattering technique, spheroplasts of the double-deletion strain expressing human AQP1, AQP3, AQP7, and AQP9 were exposed to a hyperosmolal solution and the change in light scattering was measured. Interestingly, spheroplasts expressing the human aquaglyceroporins still exhibit poor water transport as evidenced by rate constants of 0.5–1.0 s^−1^, which are comparable to the background strain (Fig. 4). On the other hand, spheroplasts expressing the orthodox AQP1 has a strikingly higher transport rate (Fig. 4a), previously estimated to be about ten times faster than passive diffusion (Fischer et al. 2009). The discrepancy between our results and the results from previous reports using *X. laevis* oocytes and model membranes, may be explained by the fact that in our system the aquaglyceroporins are in native cell membranes. In fact, water transport has previously been investigated in native membranes for the three investigated aquaglyceroporins. For instance, after deletion of the *AQP3* gene in human erythrocytes, no reduction in water transport was detected (Roudier et al. 2002), similarly deletion of the *AQP7* gene in kidney brush-border membranes made into vesicles, showed osmotic water permeability in the same range as published for AQP7 expressed in *Xenopus* oocytes (*P*_f_ ~ 0.015 cm/sec) (Geyer et al. 2013; Kondo et al. 2002; Sohara et al. 2005). Finally, after deletion of *AQP9* gene in erythrocytes, there was no detectable effect of water permeability (Liu et al. 2007) and along those lines, rat AQP3 and rat AQP9 and another aquaglyceroporin from *Plasmodium falciparum*, PfAQP, were evaluated for water activity when expressed in the native lipid bilayer in the yeast *Saccharomyces cerevisiae*, and showed to have comparable water transport to that of passive diffusion (Hedfalk et al. 2008; Pettersson et al. 2006). Taken together, these data strongly support the notion that human aquaglyceroporins have very low water transport activity when expressed in native membranes; comparable to the passive diffusion of water over the plasma membrane (Figs. 3, 4). However, there are reports from rodent knock-out models and cell lines showing deletion of at least mouse AQP3 could affect the water transport activity in mouse cells, indicating that there may be differences between species (Hara-Chikuma and Verkman 2008a).

### The Human Aquaglyceroporins Facilitate the Transport of Glycerol

**Fig. 5:**
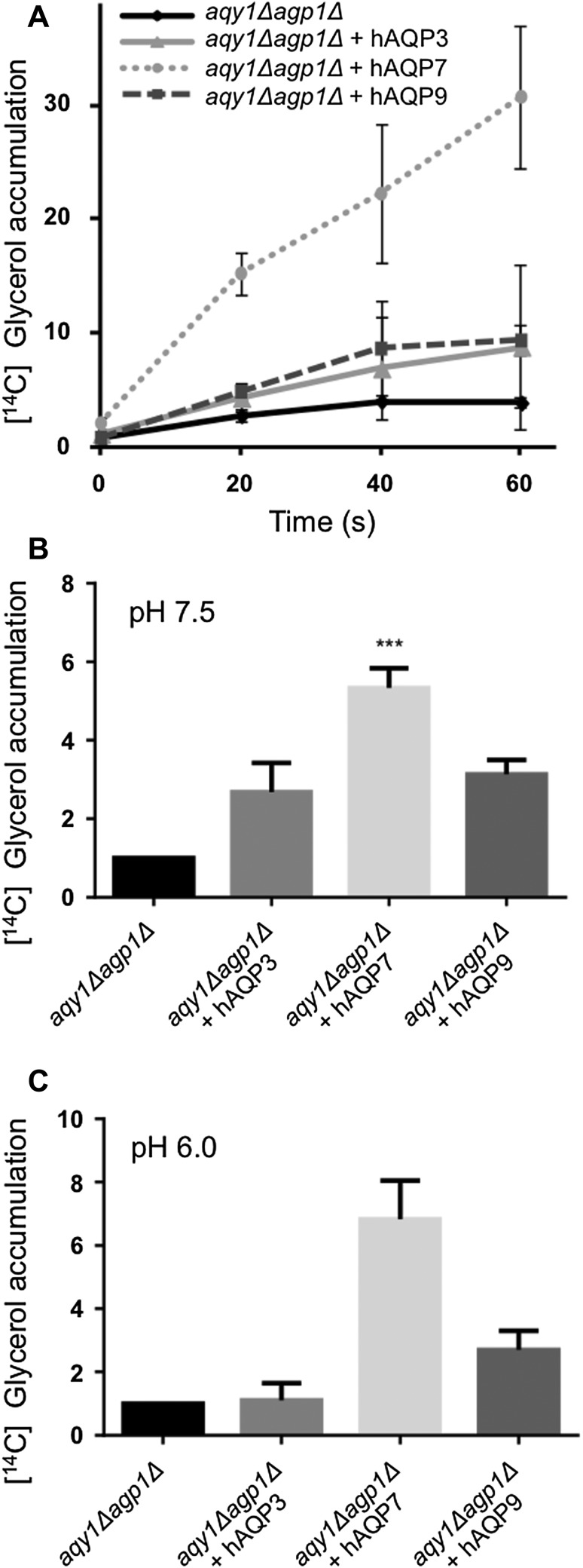
The human aquaglyceroporin are efficient glycerol facilitators. Human aquaglyceroporins overexpressed in *aqy1Δagp1Δ* double-deletion strain were tested for their glycerol transport properties at two different pHs by supplementing a mix of glycerol and ^14^C glycerol. **a** At pH 7.5, cells were exposed to glycerol and the accumulation plotted at different time points. The relative glycerol uptake per OD unit is plotted (normalized to the mean 0-value in each experiment, and error bars shows SD). **b** Relative glycerol uptake per OD unit at 30 s (pH 7.5), values are mean of triplicates normalized to *aqy1Δagp1Δ* in each experiment, and error bars denote SEM. **c** Relative glycerol uptake per OD unit at 30 s (pH 6.0), values are mean of duplicates normalized to *aqy1Δagp1Δ* in each experiment, and error bars denote SD

The main substrate for aquaglyceroporins is known to be glycerol and the three human aquaglyceroporins AQP3, AQP7, and AQP9 have all previously been shown to transport glycerol in *X. laevis* oocytes (Chauvigne et al. 2011; Kondo et al. 2002; Ohgusu et al. 2008). In our system, we detected water transport rates for the human aquaglyceroporins at levels comparable to that of passive diffusion. To confirm that the *P. pastoris* system does not negatively influence the transport capability of the proteins in general, glycerol accumulation was measured for the different human aquaglyceroporins. At pH 7.5, glycerol accumulation was observed for all strains expressing the aquaglyceroporins although with somewhat varying efficiency (Fig. 5a, b). AQP7 was the most pronounced glycerol facilitator, whereas cells expressing AQP3 and AQP9 only accumulated approximately half the glycerol amount compared to AQP7 (Fig. 5b). At pH 6.0, AQP3 lost its glycerol transport ability (Fig. 5c), which is consistent with earlier observations (Zeuthen and Klaerke 1999), and also confirm proper functionality of the human AQPs in the yeast system. The transport efficiency of AQP7 and AQP9 was as expected, not affected by the change in pH (Fig. 5c). Thus, all the aquaglyceroporins are functional glycerol channels when expressed in the double-deletion *P. pastoris* strain, and hence dysfunctional proteins cannot explain the low water uptake.

### AQP7 and AQP9 are Potent Arsenic Trioxide Facilitators

**Fig. 6:**
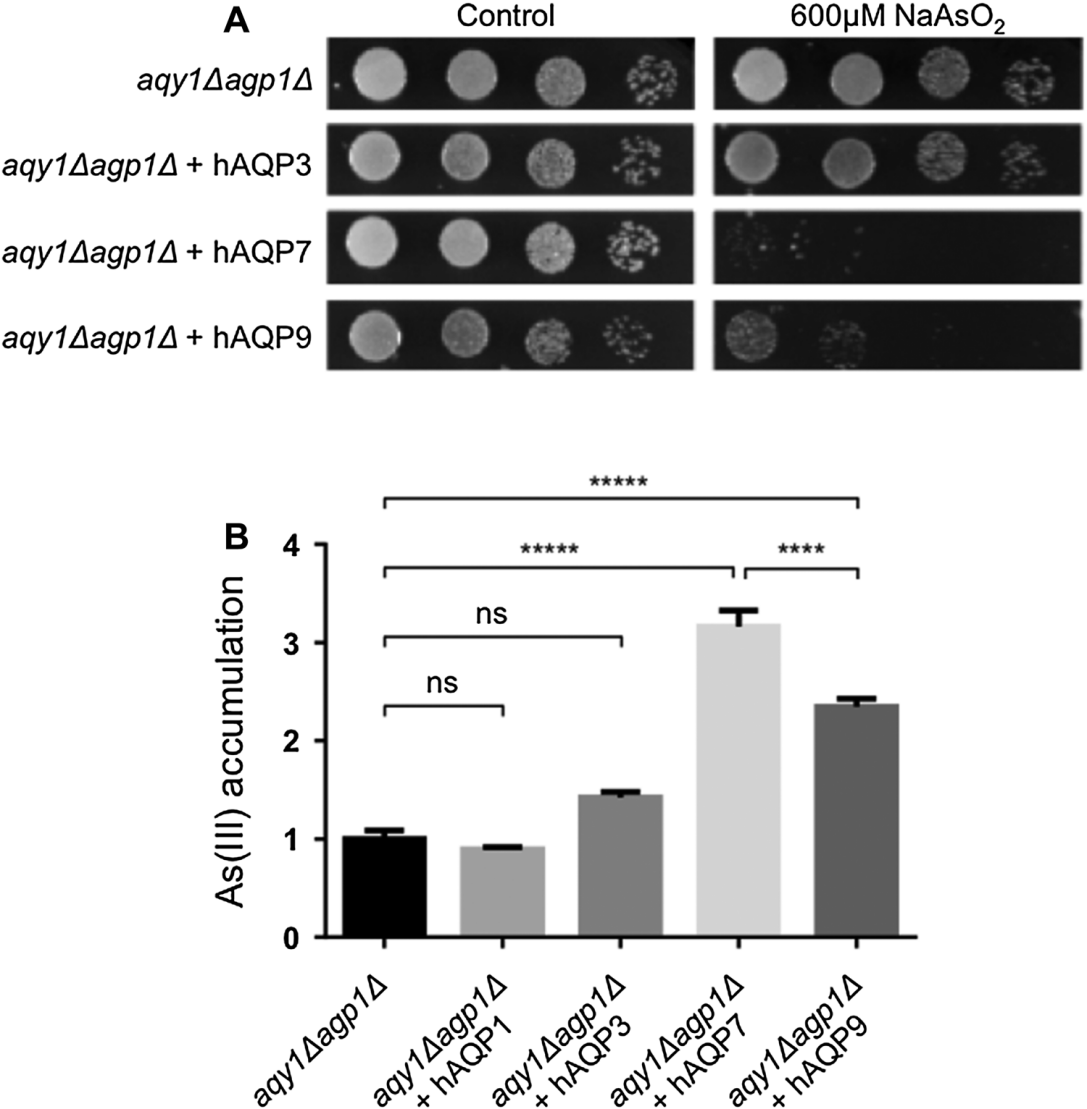
Human AQP7 and AQP9 are efficient As(III) facilitators. **a** Cells are spotted in 10-time dilution series on Control (BMMY, Buffered Methanol complex Media) and with NaAsO_2_ supplemented. **b** Accumulated As(III) concentrations at *t* = 300 s the presence of 100 mM NaAsO_2_ determined using ICP-MS. Values are mean of triplicates normalized to *aqy1Δagp1Δ* in each experiment, and error bars denote SEM

The aquaglyceroporins are also known to facilitate the transport of arsenic trioxide, As(III), and As(III) accumulation has previously been measured for the human aquaglyceroporins using *X. laevis* oocytes (Liu et al. 2004) and in another yeast, *Saccharomyces cerevisiae* (Liu et al. 2002). In both systems, human AQP7 and AQP9 were concluded to be efficient As(III) facilitators, while AQP3 was shown to be a poor As(III) facilitator in *X. leavis* oocytes. In the *P. pastoris* double-deletion strain, cells expressing AQP7 and AQP9 had difficulties to survive at high concentrations of As(III), indicating that AQP7 and AQP9 are As(III) facilitators (Fig. 6a), which is in accordance with previous findings (Liu et al. 2002, 2004). Cells expressing AQP3 were, however, unaffected, suggesting that AQP3 is a poor facilitator of As(III) (Fig. 6a), which is also consistent with previously data from the *X. laevis* oocyte system (Liu et al. 2004). To investigate the uptake rates of As(III), the intracellular As(III) levels were measured using inductively coupled plasma mass spectrometry (ICP-MS). In agreement with the growth assays, cells expressing human AQP7 and AQP9 accumulated As(III), while cells expressing AQP3 did not significantly accumulate As(III) compared to the double AQP deletion strain (Fig. 5b). These results show that AQP7 and AQP9 are much more potent As(III) facilitators compared to AQP3, which correlates well with the growth phenotypes (Fig. 6a). AQP1 was included as a negative control and did not facilitate transport of As(III) (Fig. 6b). In addition, AQP7 was shown to exhibit significantly higher arsenic trioxide transport efficiency than AQP9 (Fig. 6b), which has recently also been confirmed in the *X. laevis* oocyte system (McDermott et al. 2010). This contradicts data presented by Liu and co-workers, where AQP9 was four times as efficient in As(III) transport compared to AQP7 (Liu et al. 2004). However, Liu and co-workers used a truncated variant of AQP7, which most likely explains why the authors detected lower As(III) transport efficiency for AQP7 (Liu et al. 2004). The finding that AQP7 is a very efficient As(III) transporter may have implications for human physiology and disease. In particular, arsenic trioxide is efficacious compound in treatment of acute promyelocytic leukemia (APL) and the expression levels of AQP9 in primary leukemia samples have been suggested to be a useful predictor for the efficacy of arsenic therapy (Leung et al. 2007). Since human AQP7 seems to be an even more efficient As(III) facilitator, the expression levels of AQP7 should also be considered in accurately evaluating the potential therapeutic benefits of arsenic trioxide in treating APL.

## Conclusions

Human aquaglyceroporins are commonly referred to as channels facilitating the transport of glycerol in addition to water. Available methods for estimating the permeability of water through AQP channels are mainly restricted to non-polarizable cells (oocytes) or model membranes. This study uses a novel yeast-based system combined with a newly developed NMR method, and suggests that the main role of aquaglyceroporins is not related to water movement, but rather transport of glycerol and arsenic trioxide across the plasma membrane. Human aquaglyceroporins are likely not able to exclude water entirely, but the transport rates are not significantly different from passive diffusion of water across the plasma membrane, and hence likely of limited physiological relevance.

## References

[CR1] Azad AK, Sawa Y, Ishikawa T, Shibata H (2009). Heterologous expression of tulip petal plasma membrane aquaporins in *Pichia pastoris* for water channel analysis. Appl Environ Microbiol.

[CR2] Boury-Jamot M, Sougrat R, Tailhardat M, Le Varlet B, Bonte F, Dumas M, Verbavatz JM (2006). Expression and function of aquaporins in human skin: is aquaporin-3 just a glycerol transporter?. Biochem Biophys Acta.

[CR3] Chauvigne F, Lubzens E, Cerda J (2011). Design and characterization of genetically engineered zebrafish aquaporin-3 mutants highly permeable to the cryoprotectant ethylene glycol. BMC Biotechnol.

[CR4] Cregg JM (2007). DNA-mediated transformation methods in molecular biology.

[CR5] Day RE (2014). Human aquaporins: regulators of transcellular water flow. Biochem Biophys Acta.

[CR6] De Schutter K (2009). Genome sequence of the recombinant protein production host *Pichia pastoris*. Nat Biotechnol.

[CR7] Eriksson S, Elbing K, Soderman O, Lindkvist-Petersson K, Topgaard D, Lasic S (2017). NMR quantification of diffusional exchange in cell suspensions with relaxation rate differences between intra and extracellular compartments. PLoS ONE.

[CR8] Fischer G (2009). Crystal structure of a yeast aquaporin at 1.15 angstrom reveals a novel gating mechanism. PLoS Biol.

[CR9] Geyer RR, Musa-Aziz R, Qin X, Boron WF (2013). Relative CO_2_/NH_3_ selectivities of mammalian aquaporins 0–9. Am J Physiol Cell Physiol.

[CR10] Hara-Chikuma M, Verkman AS (2008). Aquaporin-3 facilitates epidermal cell migration and proliferation during wound healing. J Mol Med (Berl).

[CR11] Hara-Chikuma M, Verkman AS (2008). Roles of aquaporin-3 in the epidermis. J Invest Dermatol.

[CR12] Hedfalk K, Pettersson N, Oberg F, Hohmann S, Gordon E (2008). Production, characterization and crystallization of the *Plasmodium falciparum* aquaporin. Protein Expr Purif.

[CR13] Ishibashi K, Kuwahara M, Gu Y, Tanaka Y, Marumo F, Sasaki S (1998). Cloning and functional expression of a new aquaporin (AQP9) abundantly expressed in the peripheral leukocytes permeable to water and urea, but not to glycerol. Biochem Biophys Res Commun.

[CR14] Kondo H (2002). Human aquaporin adipose (AQPap) gene: genomic structure, promoter analysis and functional mutation. Eur J Biochem/FEBS.

[CR15] Laforenza U, Bottino C, Gastaldi G (2016). Mammalian aquaglyceroporin function in metabolism. Biochem Biophys Acta.

[CR16] Leung J, Pang A, Yuen WH, Kwong YL, Tse EW (2007). Relationship of expression of aquaglyceroporin 9 with arsenic uptake and sensitivity in leukemia cells. Blood.

[CR17] Liu Z, Shen J, Carbrey JM, Mukhopadhyay R, Agre P, Rosen BP (2002). Arsenite transport by mammalian aquaglyceroporins AQP7 and AQP9. Proc Natl Acad Sci U S A.

[CR18] Liu Z, Carbrey JM, Agre P, Rosen BP (2004). Arsenic trioxide uptake by human and rat aquaglyceroporins. Biochem Biophys Res Commun.

[CR19] Liu Y (2007). Aquaporin 9 is the major pathway for glycerol uptake by mouse erythrocytes, with implications for malarial virulence. Proc Natl Acad Sci U S A.

[CR20] Maeda N (2012). Implications of aquaglyceroporins 7 and 9 in glycerol metabolism and metabolic syndrome. Mol Asp Med.

[CR21] McDermott JR, Jiang X, Beene LC, Rosen BP, Liu Z (2010). Pentavalent methylated arsenicals are substrates of human AQP9. Biometals.

[CR22] Muller-Lucks A, Gena P, Frascaria D, Altamura N, Svelto M, Beitz E, Calamita G (2013). Preparative scale production and functional reconstitution of a human aquaglyceroporin (AQP3) using a cell free expression system. N Biotechnol.

[CR23] Ohgusu Y (2008). Functional characterization of human aquaporin 9 as a facilitative glycerol carrier. Drug Metab Pharmacokinet.

[CR24] Panaretou B, Piper P (2006). Isolation of yeast plasma membranes. Methods Mol Biol.

[CR25] Pettersson N, Hagstrom J, Bill RM, Hohmann S (2006). Expression of heterologous aquaporins for functional analysis in *Saccharomyces cerevisiae*. Curr Genet.

[CR26] Roudier N, Ripoche P, Gane P, Le Pennec PY, Daniels G, Cartron JP, Bailly P (2002). AQP3 deficiency in humans and the molecular basis of a novel blood group system, GIL. J Biol Chem.

[CR27] Sohara E, Rai T, Miyazaki J, Verkman AS, Sasaki S, Uchida S (2005). Defective water and glycerol transport in the proximal tubules of AQP7 knockout mice. Am J Physiol Renal Physiol.

[CR28] Tamas MJ (1999). Fps1p controls the accumulation and release of the compatible solute glycerol in yeast osmoregulation. Mol Microbiol.

[CR29] Ternes P (2011). Two pathways of sphingolipid biosynthesis are separated in the yeast *Pichia pastoris*. J Biol Chem.

[CR30] Verkman AS (2012). Aquaporins in clinical medicine. Annu Rev Med.

[CR31] Zeuthen T, Klaerke DA (1999). Transport of water and glycerol in aquaporin 3 is gated by H^+^. J Biol Chem.

